# Inspiratory- and expiratory-gated transcutaneous vagus nerve stimulation have different effects on heart rate in healthy subjects: preliminary results

**DOI:** 10.1007/s10286-019-00604-0

**Published:** 2019-04-02

**Authors:** Bartłomiej Paleczny, Rafał Seredyński, Beata Ponikowska

**Affiliations:** 1 Department of Physiology, Wroclaw Medical University, Chalubińskiego 10, 50-368 Wrocław, Poland; 2 Department of Cardiology, Centre for Heart Diseases, 4th Military Hospital, Wrocław, Poland

**Keywords:** Transcutaneous auricular vagus nerve stimulation, Respiratory-gated, Baroreflex sensitivity

## Abstract

**Purpose:**

Transcutaneous auricular vagus nerve stimulation (taVNS) has been considered for the treatment of sympathetically mediated disorders. However, the optimal mode of stimulation is unknown. This study aimed to compare the cardiovascular effects of respiratory-gated taVNS in healthy subjects.

**Methods:**

The examination included expiratory-gated, inspiratory-gated, and non-respiratory-gated taVNS trials. Subjects were examined twice (the order of expiratory- and inspiratory-gated taVNS was changed). taVNS trials started with controlled breathing without stimulation (pre-stimulatory recording) followed by controlled breathing with taVNS (stimulatory recording). Synchronizing taVNS with the respiratory phase was computer-controlled. Heart rate (HR) was calculated from ECG. Systolic blood pressure (SBP) and systemic vascular resistance (SVR) were recorded continuously and noninvasively. Baroreflex sensitivity based on rising (BRS-UP) or falling SBP sequences (BRS-DOWN) or all sequences (BRS-ALL) and heart rate variability (HRV) were analyzed.

**Results:**

Seventy-two taVNS trials were obtained from 12 subjects (age 23 ± 3 years). Pre-stimulatory HR correlated with change in HR (*r* = − 0.25) and SVR (*r* = 0.24, both *p* < 0.05). There were no differences between three stimulatory conditions in (1) the changes of hemodynamic parameters, (2) BRS-UP and BRS-ALL, or (3) HRV indices (all *p* > 0.20). However, in the group of high pre-stimulatory HR trials, HR change differed between inspiratory-gated (0.11 ± 0.53%) and both expiratory-gated (− 1.30 ± 0.58%, *p* = 0.06) and non-respiratory-gated taVNS (− 1.69 ± 0.65, *p* = 0.02). BRS-DOWN was higher in inspiratory- vs. non-respiratory-gated taVNS (15.4 ± 1.3 vs. 14.1 ± 0.9 ms/mmHg, *p* = 0.03).

**Conclusions:**

Expiratory-gated and non-respiratory-gated taVNS exert clear cardioinhibitory effects in healthy subjects with high pre-stimulatory HR, whereas inspiratory-gated taVNS does not affect HR. Cardiac and vascular effects of taVNS depend on pre-stimulatory HR.

## Introduction

Autonomic imbalance with increased sympathetic and decreased parasympathetic drive to the heart and blunted cardiac baroreflex sensitivity (BRS) are hallmarks of heart failure that may contribute to the pathogenesis and progression of the disease [1]. Consequently, novel therapies targeting the autonomic nervous system have been proposed, including electrical stimulation of cervical vagus nerve (vagus nerve stimulation, VNS) and carotid baroreceptors (baroreflex activation therapy, BAT) with implantable pacemaker-like devices [2]. However, given that both the surgical procedure and the electrostimulation itself carry a risk of adverse effects [3], the method of noninvasive, transcutaneous stimulation of the auricular branch of the vagus nerve (taVNS) located medial of the tragus at the entry of the acoustic meatus has been developed [4].

Recent studies in healthy volunteers have demonstrated that taVNS acutely improves BRS [5], decreases muscle sympathetic nerve activity [6], and favorably affects heart rate variability (HRV) [5–7]. However, optimal parameters of taVNS remain undetermined [8].

In the current study, we addressed the physiological rationale for coupling taVNS to the phase of the respiratory cycle directly by comparing the cardiovascular consequences of inspiratory- vs. expiratory-gated taVNS in a group of healthy subjects. Activity of cardiovagal fibers changes with the respiratory cycle, being suppressed during inhalation and stimulated during exhalation [9]. Therefore, it is likely that taVNS exerts its cardioinhibitory effects during expiration, not inspiration. Moreover, taVNS was shown to produce unexpected widespread activation of nucleus tractus solitarius in humans [10] which may suggest that taVNS affects the heart function via multiple mechanisms, possibly including both cardioinhibitory and cardioexcitatory pathways. If this is true, gating the taVNS to the expiratory phase should favor the cardioinhibitory mechanisms. Furthermore, given that noxious and non-noxious cutaneous stimulation (mechanical or thermal) was demonstrated to induce heart rate acceleration [11, 12], a net effect of taVNS administrated during inspiratory phase on the heart rate would be cardioexcitatory, due to the lack of concomitant inhibitory influence of the vagus nerve activity. Following this reasoning, we expected that the physiological responses to the expiratory-gated taVNS (i.e., decrease in heart rate and increase in BRS and vagally mediated HRV) would be greater as compared with the inspiratory-gated taVNS or continuous (non-respiratory-gated) taVNS.

We also hypothesized that the physiological response to artificial activation of the vagus nerve with taVNS would be greater in subjects with initially low vagal drive (manifested by the high baseline heart rate) as compared with the subjects displaying relatively high vagal drive at the start of the experiment. Therefore, the effect of interaction between taVNS stimulatory mode and pre-stimulatory heart rate was included in the analysis.

## Methods

### Study population

Healthy subjects participated in the study. The study protocol was approved by the local institutional ethics committee. Each subject gave his/her informed written consent prior to participation. The study was conducted according to the Declaration of Helsinki.

### Measurement equipment

The electrocardiogram was recorded (BioAmp, ADInstruments, New Zealand) and heart rate (HR, bpm) was calculated from the ECG signal. Arterial blood pressure was recorded continuously and noninvasively using the Nexfin monitoring device (BMEYE, Amsterdam, Netherlands). Systolic (SBP, mmHg) and mean arterial (MAP, mmHg) blood pressure and systemic vascular resistance (SVR, dyn s/cm^5^) were derived from the Nexfin device.

The subject breathed through the mask (Hans Rudolph, Inc., USA) and a two-way non-rebreathing valve (Hans Rudolph, Inc.). Differential pressure transducers (FE141, Spirometer, ADInstruments) with a flowhead (MLT3000L, ADInstruments) positioned on the inspiratory side of the valve were used to record inspiratory airflow. Minute ventilation (VI, L/min) was calculated as a product of instantaneous values of tidal volume (VT, L) and breathing rate (BR, breaths/min).

All data were acquired at 1000 Hz using a data acquisition system (PowerLab, ADInstruments) and stored on PC.

### Breathing control procedure

Respiratory activity is known to influence the heart rate [13]. Therefore, to control the respiratory-related variations in the heart rate, the controlled breathing approach was used. The breathing control procedure has been described in detail previously [14]. During the taVNS trials, the subject was instructed to adjust (1) the depth of breathing to the visual cue and (2) the rate of breathing to the auditory cue. The visual cue for controlling the depth of breathing was a real-time inspiratory airflow tracing recorded continuously with the study equipment and displayed on the computer monitor positioned above the subject’s head. The VT was pre-specified at 120% of the baseline value and the upper and lower limits were constructed as 120 + 5% and 120 − 5% of the baseline values, respectively, and were displayed on the monitor along with the inspiratory airflow. The subject was instructed to keep the peak of each inspiration between the upper and lower limits.

The auditory cue for controlling the rate of breathing was emitted by the online metronome (https://www.virtualsheetmusic.com/metronome/) and included two accents, indicating the onset of inspiration and expiration. Minor adjustments in the VT limits and/or the metronome rate were made as needed until the subject confirmed that breathing was comfortable.

### Study protocol

**Fig. 1:**
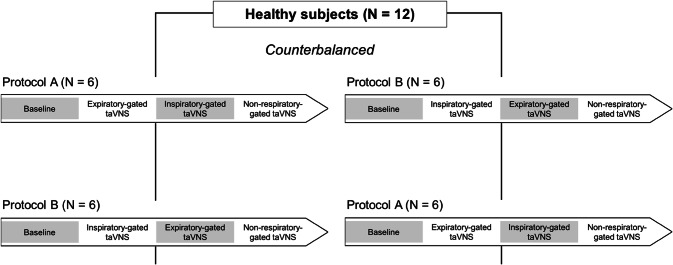
Schematic representation of the study design. taVNS, transcutaneous auricular vagus nerve stimulation

Each subject underwent two protocols (A and B) on separate days. Both protocols included (in the following order): (1) baseline recording, (2) expiratory-gated taVNS trial (protocol A) or inspiratory-gated taVNS trial (protocol B), (3) inspiratory-gated taVNS trial (protocol A) or expiratory-gated taVNS trial (protocol B), (4) non-respiratory-gated taVNS trial. The only difference between protocol A and B was the order of expiratory-gated and inspiratory-gated taVNS (either the expiratory- or the inspiratory-gated taVNS came first). The order in which the subject underwent protocols A and B was counterbalanced (Fig. 1).

The study was carried out with the subject lying supine. After the monitoring equipment was attached the subject rested for at least 15 min and the last 10 min of the recording (baseline recording) was used to calculate baseline HR, SBP, MAP, SVR, VI, VT, and BR (arithmetic means). Adjustment of the amplitude of taVNS and familiarization with the breathing control procedure were performed immediately after the baseline recording and prior to the next phase (either expiratory- or inspiratory-gated trial). All taVNS trials (expiratory- and inspiratory-gated and non-respiratory-gated) started with a 1-min period of controlled breathing without stimulation (pre-stimulatory recording) followed by a 2-min period of controlled breathing with concomitant taVNS (stimulatory recording). All trials were separated by 3–5 min.

### Transcutaneous auricular vagus nerve stimulation

**Fig. 2:**
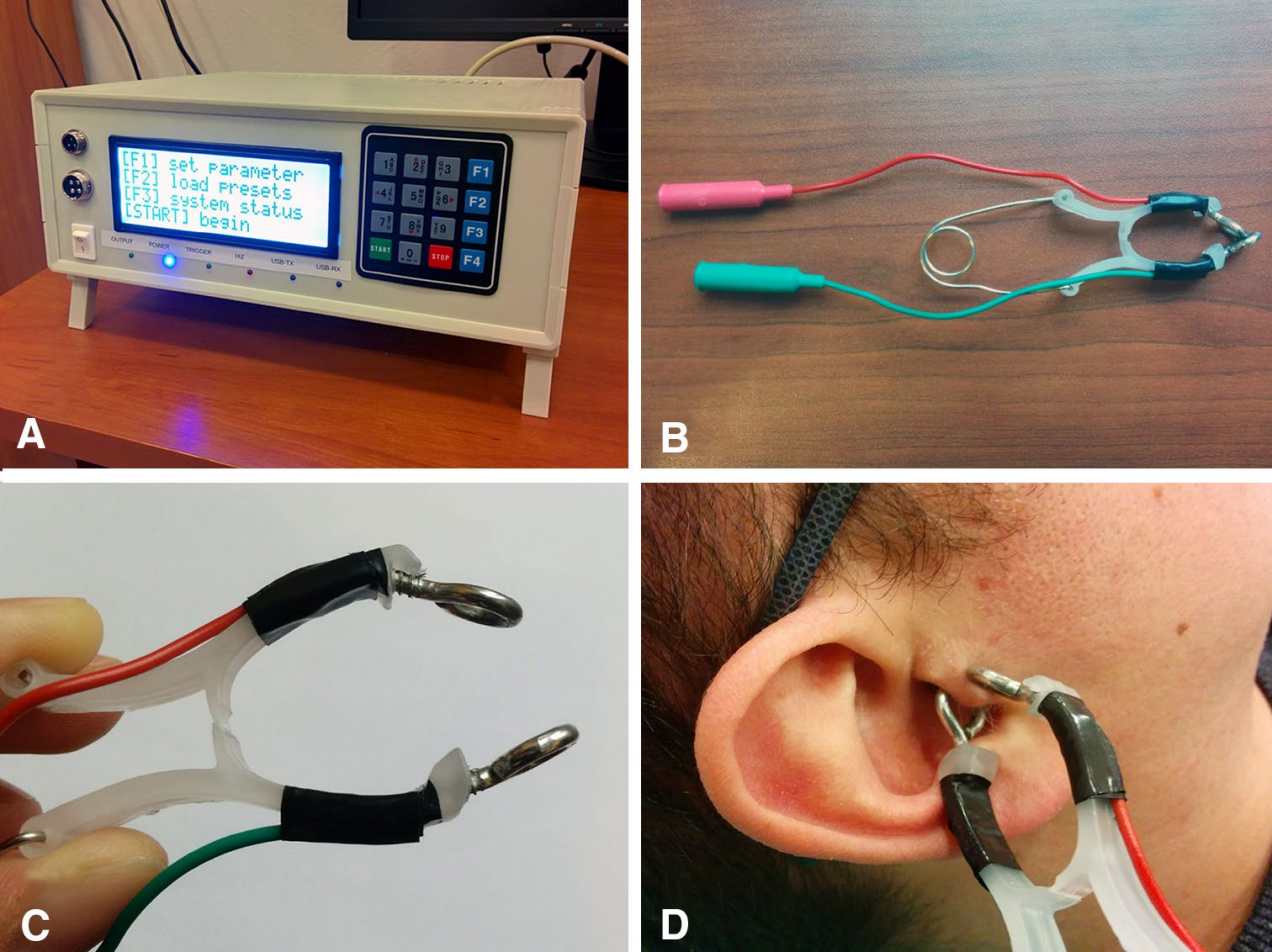
Photographs of the prototype electrostimulator (**a**) and a custom-built electrode (**b**–**d**) used in the present study

The prototype electrostimulator (IMER Systems, Wroclaw, Poland, Fig. 2a) and a custom-made electrode (Fig. 2b–d) were used for taVNS. The electrical stimulus consisted of a continuous train of rectangular, biphasic, symmetrical pulses (1000 µs/phase, interphase interval 30 µs) delivered at 25 Hz. The intensity (amplitude) of electrical stimulus was adjusted according to the subject’s sensory and pain thresholds, using a procedure similar to that of Yakunina et al. [15] and De Couck et al. [7]. In brief, the intensity of the stimulus was slowly increased from 0 by 10 µA increments until the subject reported feeling sensation (sensory threshold). Then the intensity of the stimulus was further increased until the subject reported feeling pain or intolerable discomfort (pain threshold). Finally, the stimulation intensity was set at 80% of the distance between sensory threshold and pain threshold.

**Fig. 3:**
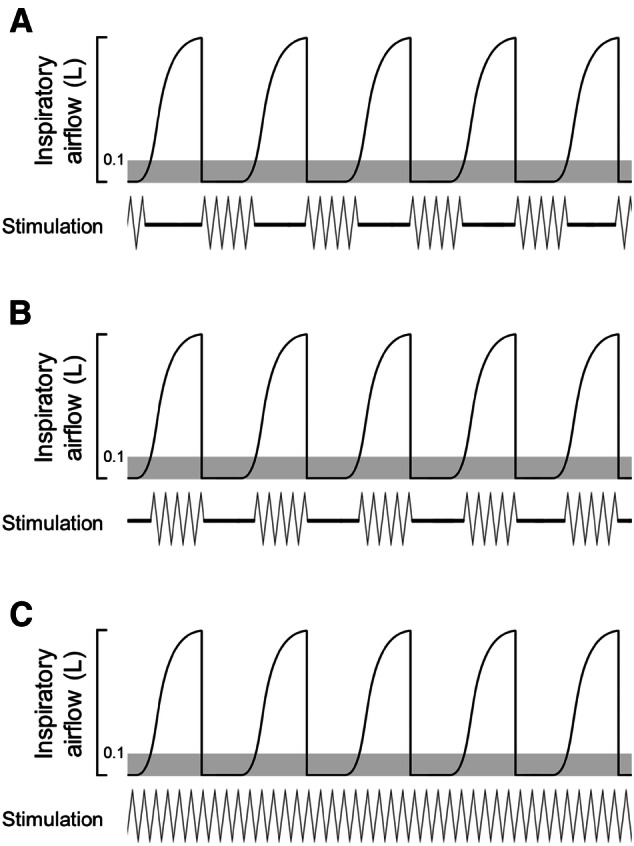
Scheme illustrating three stimulatory modes used in the present study: **a** expiratory-gated transcutaneous auricular vagus nerve stimulation (taVNS), **b** inspiratory-gated taVNS, and **c** non-respiratory-gated (continuous) taVNS

Synchronizing the stimulation with the inspiratory or expiratory phase was controlled by the LabChart software (ADInstruments) and executed by the PowerLab device connected to the electrostimulator via a digital output port. In the inspiratory-gated trials, an inspiratory airflow increase above 0.1 L turned the stimulation on, and the stimulation continued until the inspiratory airflow fell below 0.1 L; then the stimulation was turned off. Similarly, in the expiratory-gated trials, the stimulation was turned on by a fall in the inspiratory airflow below 0.1 L, and turned off by an increase in the inspiratory airflow above 0.1 L. In the non-respiratory-gated trials, continuous stimulation was applied (Fig. 3).

### Evaluation of cardiac baroreflex sensitivity

BRS was evaluated with the sequence method [16]. In brief, all sequences of at least three heart beats in which (1) SBP increases (by at least 1 mmHg) were followed by progressive lengthening in RR interval (by at least 5 ms) (UP sequences) or (2) SBP decreases (by at least 1 mmHg) were followed by progressive shortening in RR interval (by at least 5 ms) (DOWN sequences) were identified and slope of the regression line relating SBP to RR interval was calculated for each sequence identified. Only sequences with *r*^2^ ≥ 0.85 were accepted. Three BRS (ms/mmHg) values were computed: BRS-UP (as the average of slopes from all UP sequences), BRS-DOWN (as the average of slopes from all DOWN sequences), and BRS-ALL (as the average of slopes from all UP and DOWN sequences). Numbers of the accepted sequences for BRS-UP, BRS-DOWN, and BRS-ALL were reported.

### Evaluation of heart rate variability

The following parameters of HRV were calculated: standard deviation of all RR intervals (SDNN, ms), the percentage of RR intervals differing by greater than 50 ms (pNN50, %), and the ratio of low-frequency HRV power (HRV spectrum within the range 0.04–0.15 Hz) to high-frequency HRV power (HRV spectrum within the range 0.15–0.4 Hz) (LF/HF ratio) [17]. Standard autoregressive methods have been used to calculate the LF/HF ratio [18].

### Data processing and statistical analysis

Data were presented as mean ± standard error (SE) or number as appropriate. General linear models for repeated measures were used to test for the effects of taVNS stimulatory condition (expiratory- vs. inspiratory-gated vs. non-respiratory-gated) and the effects of interaction: taVNS stimulatory condition × pre-stimulatory HR. Fisher’s least significant difference (LSD) test was used as the post-hoc. Paired Student’s *t* test was used to test differences in baseline parameters between protocol A vs B. Pearson’s correlation coefficient was used to test the linear relationship between examined variables.

A 1-min period of controlled breathing without stimulation (pre-stimulatory recording) was used to calculate pre-stimulatory HR, SVR, SBP, and MAP (arithmetic means). The first minute of stimulatory recording (controlled-breathing with concomitant taVNS) was used to calculate stimulatory HR, SVR, SBP, and MAP (arithmetic means). Differences between pre- and stimulatory values of hemodynamic parameters were expressed as percentages of pre-stimulatory values. Positive values indicate an increase in a given parameter from pre- to stimulatory period, while negative values indicate a decrease in the parameter.

BRS and HRV indices were calculated from the entire 2-min period of controlled breathing with concomitant taVNS (stimulatory recording).

The median value of pre-stimulatory HR for all non-respiratory taVNS trials was used to classify the examination into a low (below median) or high (equal or above median) pre-stimulatory HR group.

SPSS v. 23.0 (SPSS Software Inc., Chicago, IL, USA) and MATLAB v. 2016a (Mathworks, Natick, MA, USA) were used for data processing and statistical analysis. CardioSeries 2.4 was used to calculate BRS (https://www.danielpenteado.com). Kubios [19] was used to calculate HRV indices. A *p* value below 0.05 was considered significant.

## Results

The study sample included 72 trials in total (24 expiratory-gated trials, 24 inspiratory-gated trials and 24 non-respiratory-gated trials) derived from 12 healthy volunteers. Baseline characteristics of the examined subjects are presented in Table 1. Mean amplitude of taVNS was 722 ± 92 µA.

**Table 1:** Baseline characteristics of the examined subjects (*N* = 12)

	Protocol A	Protocol B	*p*
Males, number (%)	6 (50)	–	–
Age, years	23 ± 3	–	–
Body mass index, kg/m^2^	23.2 ± 3.5	–	–
HR, bpm	71 ± 4	72 ± 3	0.34
SBP, mmHg	120 ± 6	122 ± 3	0.85
MAP, mmHg	89 ± 4	88 ± 2	0.73
SVR, dyn s/cm^5^	992 ± 76	898 ± 43	0.11
VI, L/min	9.4 ± 0.6	9.0 ± 0.5	0.50
VT, L	0.78 ± 0.09	0.72 ± 0.07	0.58
BR, breaths/min	13 ± 1	13 ± 1	0.66

Data are presented as mean ± SE; *p* values for paired *t* test are presented

*HR* heart rate,* SBP* systolic blood pressure,* MAP* mean arterial blood pressure,* SVR* systemic vascular resistance,* VI* minute ventilation,* VT* tidal volume,* BR* breathing rate

### Relations between pre-stimulatory heart rate and hemodynamic effects of taVNS

**Fig. 4:**
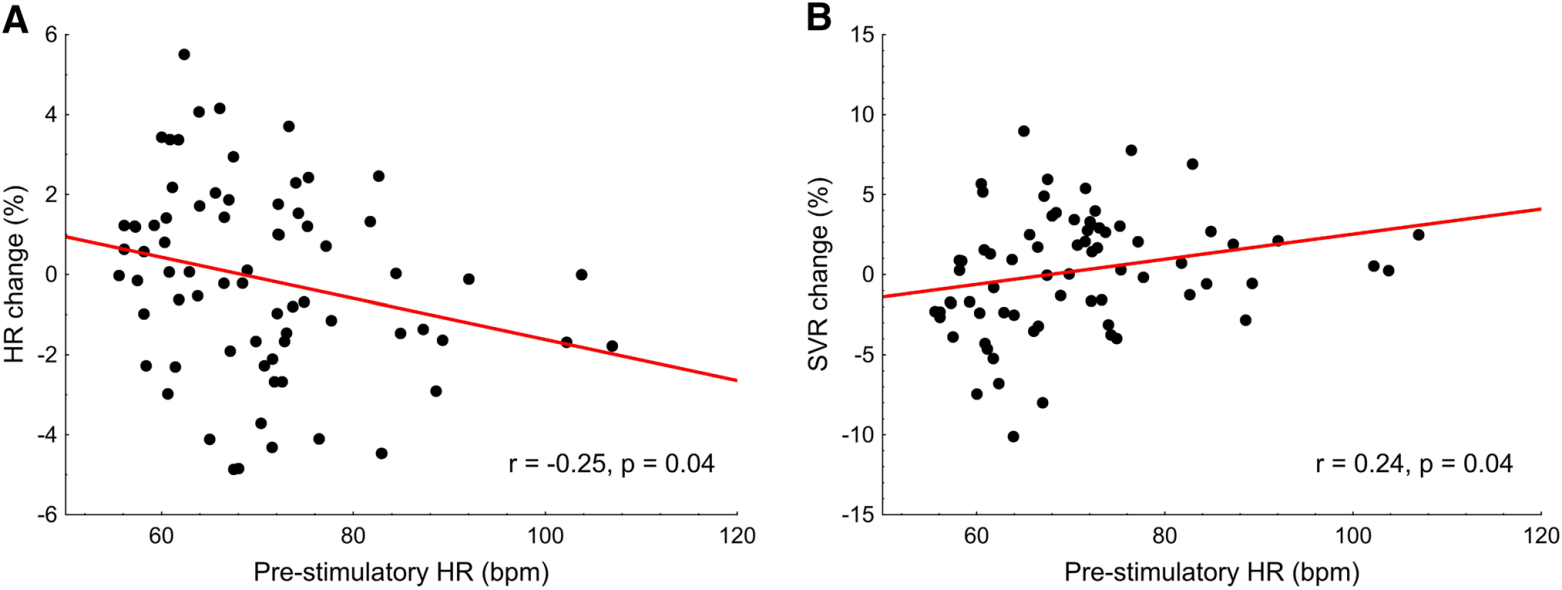
Correlations between pre-stimulatory HR and **a** HR change and **b** SVR change for all trials analyzed (*N* = 72)

Pre-stimulatory HR correlated with the change in HR (*r* = − 0.25, *p* = 0.04, Fig. 4a) and SVR (*r* = 0.24, *p* = 0.04, Fig. 4b), but not with the changes in SBP and MAP (both *p* > 0.40).

### Differences in hemodynamic effects of taVNS between expiratory-gated, inspiratory-gated, and non-respiratory-gated trials

**Fig. 5:**
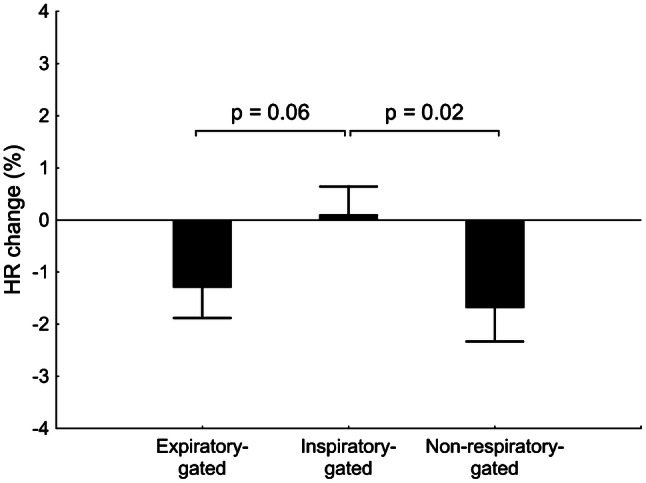
Percentage changes in HR across three stimulatory conditions for trials with pre-stimulatory HR ≥ 67 bpm. *p* values for Fisher’s LSD post hoc test are presented

The analysis using general linear model for repeated measures revealed a significant effect of the interaction between taVNS stimulatory condition and pre-stimulatory HR on the HR response (*p* = 0.04, Table 2). Post hoc comparisons identified significant differences in high pre-stimulatory HR group [pre-stimulatory HR ≥ 67 bpm (median)] between inspiratory-gated taVNS (0.11 ± 0.53%) and both expiratory-gated taVNS (− 1.30 ± 0.58%, *p* = 0.06) and non-respiratory-gated taVNS (− 1.69 ± 0.65%, *p* = 0.02, Fig. 5). There were no other significant differences (all *p* ≥ 0.13). The results indicate that expiratory-gated and non-respiratory-gated taVNS decrease HR, while inspiratory-gated taVNS has no effect on HR and this is restricted to subjects with relatively high pre-stimulatory HR level. These results corroborate the relation between pre-stimulatory HR and taVNS-induced change in HR (Fig. 4a).

**Table 2:** Percentage changes in hemodynamic parameters across three stimulatory conditions employed

	Expiratory-gated taVNS	Inspiratory-gated taVNS	Non-respiratory-gated taVNS	Stimulatory condition *p* value	Interaction: stimulatory condition × pre-stimulatory HR *p* value
HR change (%)					
All trials	0.13 ± 0.50	0.23 ± 0.39	− 0.68 ± 0.53	0.23	0.04
Pre-stimulatory HR < 67 bpm	1.82 ± 0.48	0.38 ± 0.60	0.52 ± 0.75		
Pre-stimulatory HR ≥ 67 bpm	− 1.30 ± 0.58	0.11 ± 0.53	− 1.69 ± 0.65		
SVR change (%)					
All trials	0.39 ± 0.85	− 0.06 ± 0.61	0.29 ± 0.82	0.94	0.13
Pre-stimulatory HR < 67 bpm	− 2.53 ± 1.20	− 0.84 ± 1.06	− 1.37 ± 1.36		
Pre-stimulatory HR ≥ 67 bpm	2.86 ± 0.66	0.61 ± 0.65	1.71 ± 0.83		
SBP change (%)					
All trials	1.00 ± 0.54	1.40 ± 0.64	0.84 ± 0.38	0.58	0.74
Pre-stimulatory HR < 67 bpm	− 0.04 ± 0.42	0.29 ± 0.53	0.13 ± 0.38		
Pre-stimulatory HR ≥ 67 bpm	1.88 ± 0.87	2.34 ± 1.04	1.43 ± 0.60		
MAP change (%)					
All trials	0.69 ± 0.45	0.88 ± 0.53	0.32 ± 0.36	0.58	0.96
Pre-stimulatory HR < 67 bpm	− 0.06 ± 0.56	− 0.04 ± 0.54	− 0.47 ± 0.48		
Pre-stimulatory HR ≥ 67 bpm	1.33 ± 0.65	1.66 ± 0.80	0.99 ± 0.47		

Data are presented as mean ± SE; *p* values for general linear model for repeated measures are presented

*taVNS* transcutaneous auricular vagus nerve stimulation,* HR* heart rate,* SVR* systemic vascular resistance,* SBP* systolic blood pressure,* MAP* mean arterial blood pressure

### Differences in cardiac baroreflex sensitivity and heart rate variability indices between expiratory-gated, inspiratory-gated, and non-respiratory-gated taVNS conditions

Mean ± SE numbers of the sequences extracted for BRS calculation were (1) 15.7 ± 0.9 (BRS-UP) and 15.1 ± 0.8 (BRS-DOWN) for expiratory-gated taVNS trials, (2) 16.2 ± 0.9 (BRS-UP) and 15.2 ± 0.8 (BRS-DOWN) for inspiratory-gated taVNS trials, and (3) 15.6 ± 0.8 (BRS-UP) and 14.9 ± 0.7 (BRS-DOWN) for non-respiratory-gated taVNS trials.

We found a marginally significant effect (*p* = 0.06) of stimulatory condition on the baroreflex sensitivity to falling blood pressure (BRS-DOWN). Post hoc comparisons revealed that BRS-DOWN in non-respiratory-gated taVNS trials (14.1 ± 0.9 ms/mmHg) was lower or tended to be lower compared with inspiratory-gated (15.4 ± 1.3 mmHg, *p* = 0.03) and expiratory-gated taVNS trials (15.1 ± 1.1 ms/mmHg, *p* = 0.10), respectively.

No other differences were found for BRS (Table 3) or HRV (Table 4) parameters.

**Table 3:** Cardiac baroreflex sensitivity across three stimulatory conditions employed

	Expiratory-gated taVNS	Inspiratory-gated taVNS	Non-respiratory-gated taVNS	Stimulatory condition *p* value	Interaction: stimulatory condition × pre-stimulatory HR *p* value
BRS-UP, ms/mmHg				0.76	0.45
All trials	18.2 ± 1.5	17.8 ± 1.8	17.4 ± 1.8		
Pre-stimulatory HR < 67 bpm	21.1 ± 2.1	21.6 ± 2.3	21.6 ± 2.8		
Pre-stimulatory HR ≥ 67 bpm	15.7 ± 1.9	14.7 ± 2.5	13.8 ± 1.9		
BRS-DOWN, ms/mmHg				0.06	0.14
All trials	15.1 ± 1.1	15.4 ± 1.3	14.1 ± 0.9		
Pre-stimulatory HR < 67 bpm	18.0 ± 1.3	19.0 ± 1.6	16.4 ± 1.2		
Pre-stimulatory HR ≥ 67 bpm	12.6 ± 1.5	12.4 ± 1.6	12.2 ± 1.2		
BRS-ALL, ms/mmHg				0.21	0.63
All trials	16.5 ± 1.2	16.7 ± 1.5	15.8 ± 1.3		
Pre-stimulatory HR < 67 bpm	19.6 ± 1.6	20.3 ± 1.3	18.7 ± 1.8		
Pre-stimulatory HR ≥ 67 bpm	13.9 ± 1.6	13.6 ± 2.0	13.1 ± 1.6		

Data are presented as mean ± SE; *p* values for general linear model for repeated measures are presented

*taVNS* transcutaneous auricular vagus nerve stimulation,* HR* heart rate,* BRS-UP* baroreflex sensitivity calculated from UP systolic blood pressure–RR interval sequences (increasing systolic blood pressure and RR interval duration),* BRS-DOWN* baroreflex sensitivity calculated from DOWN systolic blood pressure–RR interval sequences (decreasing systolic blood pressure and RR interval duration),* BRS-ALL* baroreflex sensitivity calculated from ALL systolic blood pressure–RR interval sequences

**Table 4:** Heart rate variability parameters across three stimulatory conditions employed

	Expiratory-gated taVNS	Inspiratory-gated taVNS	Non-respiratory-gated taVNS	Stimulatory condition *p* value	Interaction: stimulatory condition × pre-stimulatory HR *p* value
SDNN, ms				0.83	0.57
All trials	73 ± 6	74 ± 7	71 ± 8		
Pre-stimulatory HR < 67 bpm	87 ± 9	92 ± 10	88 ± 12		
Pre-stimulatory HR ≥ 67 bpm	57 ± 8	61 ± 6	58 ± 8		
pNN50, %				0.88	0.10
All trials	34 ± 4	33 ± 4	33 ± 4		
Pre-stimulatory HR < 67 bpm	44 ± 4	49 ± 5	46 ± 4		
Pre-stimulatory HR ≥ 67 bpm	25 ± 5	20 ± 5	22 ± 5		
LF/HF ratio				0.65	0.67
All trials	4.1 ± 1.5	4.5 ± 1.4	5.2 ± 1.9		
Pre-stimulatory HR < 67 bpm	3.6 ± 1.4	4.8 ± 2.0	4.8 ± 2.1		
Pre-stimulatory HR ≥ 67 bpm	4.6 ± 2.5	4.2 ± 2.0	5.8 ± 3.1		

Data are presented as mean ± SE; *p* values for general linear model for repeated measures are presented

*taVNS* transcutaneous auricular vagus nerve stimulation,* HR* heart rate,* SDNN* standard deviation of all RR intervals,* pNN50* percentage of pairs of adjacent RR intervals differing by more than 50 ms,* LF/HF ratio* the ratio of low-frequency heart rate variability power to high-frequency heart rate variability power

## Discussion

To our knowledge, we are the first to show that expiratory-gated and inspiratory-gated taVNS exert different effects on the heart rate in healthy young volunteers. Furthermore, we found that cardiac effects of taVNS depend on pre-stimulatory heart rate level. This may explain discordant results from past studies.

### Cardiovascular effects of respiratory-gated taVNS

We found that if initial (pre-stimulatory) heart rate is relatively high, both expiratory-gated taVNS and non-respiratory-gated (continuous) taVNS are likely to decrease heart rate, whereas inspiratory-gated taVNS exerts negligible (and stimulatory rather than inhibitory) effect on the heart rate. These results confirm, in part, our hypothesis based on the observation that activity in cardiovagal fibers is suppressed during inhalation and stimulated during exhalation [9] and corroborate the findings reported by Garcia et al. [20] in migraine patients and healthy controls that expiratory-gated taVNS causes greater activation of nucleus tractus solitarius than inspiratory-gated taVNS.

The magnitude of heart rate reduction under taVNS in the present study (expiratory-gated and non-respiratory-gated taVNS for trials with high pre-stimulatory heart rate − 1.30 ± 0.85% and − 1.69 ± 0.65%, respectively) is slightly lower than that reported by Antonino et al. [5] (mean ± SE, − 3.4 ± 1%) and Badran et al. [8] (mean ± SE, − 3.13 ± 0.55 bpm). Nevertheless, all the studies in this field suggest that the effect of taVNS on heart rate is rather slight in healthy subjects.

We are aware of only two studies employing the respiratory-gated taVNS to target autonomic nervous system, both from Riccardo Barbieri’s laboratory [21, 22]. The authors reported that the expiratory-gated taVNS improved HRV in 12 hypertensive patients, e.g., as seen by increased high-frequency HRV and decreased low-frequency HRV. Unfortunately, the authors did not include the inspiratory-gated taVNS or non-respiratory-gated taVNS, thereby not allowing conclusions on the superiority of one stimulation mode over the other to be drawn. Furthermore, neither HR nor the other cardiovascular variables were presented, thus both publications provide little information on the overall cardiovascular consequences of the taVNS.

We found no difference in cardiovascular effects between expiratory-gated taVNS and non-respiratory-gated taVNS. However, given that the effect of inspiratory-gated taVNS on the heart rate seems to be rather stimulatory than inhibitory, one may speculated that expiratory-gated taVNS should be considered for clinical application. Gating the taVNS to the respiratory cycle requires continuous monitoring of respiratory activity. Conventional respiration measurement approaches, including chest-belts or oronasal probes, are inconvenient to use. However, the latest progress in physiological measurement technology led to the development of novel, less obtrusive approaches based on standard one-lead ECG tracing [23], pulse oximetry [24], or thermal imaging [25] that allow for the real-time monitoring of respiration. These approaches may be potentially incorporated into hand-held devices designed to deliver taVNS (e.g., NEMOS^®^, cerbomed, Erlangen, Germany [26]). However, none of the previous papers directly compared the cardiovascular effects of respiratory-gated and non-respiratory-gated taVNS, and clearly further studies are needed to determine the superiority of one stimulation mode over the other.

Stimulation of the carotid baroreflex by neck suction in humans [27] or direct electrostimulation of the carotid sinus nerve in anesthetized dogs [28] was demonstrated to exert maximal cardioinhibitory effects when the stimulation was applied during expiration, whereas inspiratory-coupled stimulation yielded minimal effects. However, we failed to find a difference between spontaneous beat-to-beat baroreflex sensitivity (BRS) measured during expiratory-gated taVNS and inspiratory-gated taVNS. Although the reason for this discrepancy is unknown, it should be noted that in both aforementioned studies BRS was assessed invasively with strong, baroreceptor-activating stimuli (e.g., neck chamber technique), whereas in the current study BRS was estimated using noninvasive methods based on spontaneous beat-to-beat oscillations in systolic blood pressure and RR interval duration. Future studies addressing the effects of respiratory-gated taVNS on BRS should consider employing invasive methods for BRS evaluation (e.g., the “gold standard” phenylephrine method [29]).

One surprising observation from our study is that BRS to falling blood pressure was lower under non-respiratory-gated taVNS conditions as compared with respiratory-gated taVNS trials. Whether this reflects an actual physiological effect (e.g., greater BRS-modulatory potential of transient vs. continuous taVNS) remains to be examined.

We did not observe any effect of taVNS conditions on HRV. This is contrary to the results of Sclocco et al. [21] and Garcia et al. [22] in hypertensive patients. However, differences in the population studied between our study and the aforementioned reports are likely to contribute to this discrepancy. Second, extremely short (2-min) ECG recordings, corresponding to taVNS duration, were used to calculate HRV indices. This might limit our ability to identify existing differences. Indeed, Antonino et al. [5] used longer, 15-min taVNS periods.

### Cardiovascular response to taVNS depends on pre-stimulatory heart rate

We found that both cardiac and vascular responses to taVNS are associated with pre-stimulatory heart rate. A negative relationship between the change in heart rate and pre-stimulatory heart rate indicates that the cardiac slowing in response to taVNS is present when initial heart rate is high (possibly due to sympathetic predominance and/or vagal withdrawal). This observation corroborates the findings by Clancy et al. [6] showing that the baseline LF/HF ratio (marker of sympathovagal balance of the heart) is correlated with the change in LF/HF ratio during taVNS.

A positive correlation between the change in systemic vascular resistance and pre-stimulatory heart rate indicates that taVNS was accompanied by peripheral vasoconstriction in the high-pre-stimulatory heart rate trials. This observation is, however, contradictory to the results of Clancy et al. [6] who found that taVNS decreases muscle sympathetic nerve activity in healthy subjects. Although we are not able to explain this discrepancy, it is possible that an increase in systemic vascular resistance in the high-pre-stimulatory heart rate trials was a compensatory response to decreased heart rate. Also, it should be emphasized that the correlations found in the current study are rather weak, indicating that less than 10% of the observed variability in the magnitude of heart rate and systemic vascular resistance changes during taVNS may be explained by the pre-stimulatory heart rate level.

Noteworthy, the aforementioned relation may help explain the discordant reports on the effects of taVNS on heart rate. It appears that the subjects in the studies showing the cardioinhibitory effects of taVNS had higher baseline heart rate (e.g., 72 bpm in Antonino et al.’s study [5]) as compared with the studies that failed to demonstrate this effect (e.g., 65 bpm in Busch et al.’s study [30]). Subject’s body position during the examination was shown to affect the sympathetic–parasympathetic balance [31]. Given that the taVNS was carried out with the subject in the sitting position in some studies [5, 7], while in others the subject was asked to lie in a supine [10] or semi-supine position [6] during the taVNS trial, the differences in body position between studies are likely to contribute to the differences in the baseline heart rate. Nevertheless, attention should be paid to the possible effects of the subject’s body position during taVNS in the future studies in this field.

### Study limitations

This paper reports some preliminary findings of the ongoing investigation. We are aware of important limitations of the present report. First, the small number of subjects included may have decreased the power of the statistical analysis and limited the ability of the study to identify existing effects of taVNS. Second, we did not include placebo or sham stimulation. However, the study was designed to investigate possible differences between three stimulatory modes rather than the effects of stimulation per se. Third, given that simple mental tasks (e.g., thinking about breathing) were shown to affect sympathovagal control of the heart [32], our results do not necessarily apply to spontaneous breathing conditions. Fourth, the length of the washout period separating the trials (3–5 min) was similar to that used by De Couck et al. [7]. The possibility that the effects of a given taVNS trial have carried over to the subsequent taVNS trial cannot be excluded. However, we attempted to balance the possible carry-over effect (for expiratory- and inspiratory-gated taVNS) by the study design with each subject examined twice (once using expiratory-gated then inspiratory-gated taVNS and once using inspiratory-gated then expiratory-gated taVNS). Therefore, we believe that the carry-over effect did not influence the comparison of the responses to expiratory- vs. inspiratory-gated taVNS, although it might affect the results for non-respiratory-gated taVNS. Fifth, BRS and HRV parameters were calculated from 2-min recordings, whereas 5-min recordings (or longer) are recommended [17].
